# The complete mitochondrial genome and phylogenetic analysis of the reticulated swellshark: *Cephaloscyllium fasciatum* Chan, 1966

**DOI:** 10.1080/23802359.2021.2015266

**Published:** 2022-01-10

**Authors:** Yan Liu, Binbin Shan, Changping Yang, Liangming Wang, Gongjun Zhang, Dianrong Sun

**Affiliations:** a Guangdong Provincial Key Laboratory of Fishery Ecology and Environment, Guangzhou, China; b South China Sea Fisheries Research Institute, Chinese Academy of Fisheries Sciences, Guangzhou, China; c Key Laboratory of South China Sea Fishery Resources Exploitation & Utilization, Ministry of Agriculture Rural Affairs, Guangzhou, China

**Keywords:** *Cephaloscyllium fasciatum*, swellsharks, mitogenome, Scyliorhinidae

## Abstract

In the present study, the complete mitogenome of *Cephaloscyllium fasciatum* has been sequenced and assembled. The complete mitochondrial genome is 16,509 bp in length and contains 37 mitochondrial genes and a control region as other fishes. The nucleotide composition of *C*. *fasciatum* mitogenome showed an anti-G bias and an excess of AT content. Furthermore, maximum-likelihood phylogenetic tree was constructed based on 13 protein-coding genes of *C*. *fasciatum* and other 15 sharks. This work will provide molecular data for studies on phylogeny and evolution in the family Scyliorhinidae.

## Introduction

Species of the genus *Cephaloscyllium* Gill 1862, also known as swellsharks. These species can be distinguished from other sharks in the family Scyliorhinidae due to the lack of labial furrows. Furthermore, the *Cephaloscyllium* has a unique ability that they could swallow either seawater or air, to inflate their stomachs to deter predation (Schaaf-Dasilva and Ebert 2008). Among these species, reticulated swellshark (*Cephaloscyllium fasciatum* Chan, 1966) is one of the two species distributed in the South China Sea (Nakaya et al. 2013). Although the *C*. *fasciatum* is a common species in the catch of trawl fishery, studies on this species are insufficient. Therefore, we sequenced the complete mitogenome sequence and performed the phylogenetic analysis of *C*. *fasciatum*, the results of the present study would provide basic information for further studies about the species.

## Materials and methods

The *C*. *fasciatum* specimen was collected from the Beibu Gulf of the South China Sea (N 107.8150°, E 19.2510°) on 9th September 2019. The specimen was identified based on morphological characters followed the identification keys of Nakaya et al. (2013). Then, muscle from the *C*. *fasciatum* specimen was collected and frozen in liquid nitrogen for DNA extraction. The sample was stored in the Key Laboratory of South China Sea Fishery Resources Exploitation & Utilization, Ministry of Agriculture Rural Affairs (Binbin Shan, shanbinbin@yeah.net) under the voucher number CF_Beibu01. Total genomic DNA was extracted using the Aidlab Genomic DNA Extraction Kit. The genome of *C*. *fasciatum* was sequenced by next-generation sequencing (Illumina HisSeq 4000). After trimming, 30.82 million clean reads were obtained. The clean reads were preliminarily assembled using *de novo* assembly by using SPAdes version 3.10.1 with k-mer size 127 (Bankevich et al. 2012). Then, we compared the assembled mitogenome with sequences of *C. umbratile* (NCBI accession no. NC_029399.1) to check the accuracy of the assembled mitochondrial genome. Furthermore, the mitogenome was annotated by MITOS2 (Bernt et al. 2013). Phylogenetic analysis was performed based on the protein-coding genes nucleotide sequences using the iq-tree.

## Results

**Figure 1.**
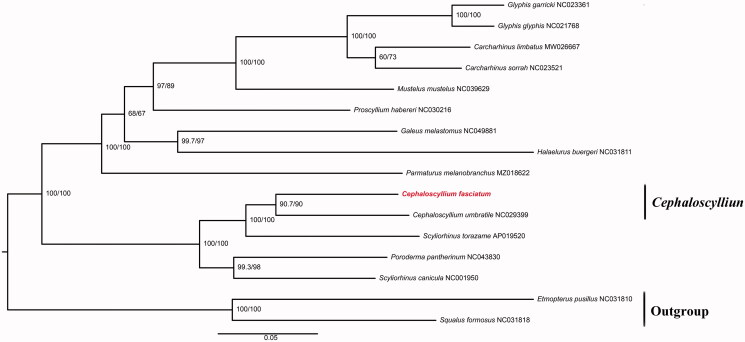
Phylogenetic tree reconstructed by maximum-likelihood (ML) analysis based on 13 protein-coding genes (PCGs) of 16 sharks, including C. fasciatum sequenced (red font) in this study. Numbers below or above branches are assessed by ML bootstrap. Numbers following scientific names are GenBank accessions.

The mitogenome of *C*. *fasciatum* is 16,703 bp in length, including a non-coding AT-rich region (D-loop region), 13 protein-coding genes, 2 ribosomal RNA (rRNA) genes, and 22 transfer RNA (tRNA) genes. The 13 protein-coding genes are 11,432 bp, accounting for 68.44% of the whole mitogenome. The nucleotide composition of *C*. *fasciatum* mitogenome is estimated to be 31.34% T, 23.58% C, 30.60% A, and 14.48% G, with anti-G bias and an excess of AT as the observation in most fishes (Miya et al. 2001). The maximum-likelihood phylogenetic tree was constructed based on 13 protein-coding genes of *C*. *fasciatum* and the other 15 sharks using the software iq-tree, *Squalus formosus*, and *Etmopterus pusillus* were selected as outgroups (Figure 1). The most suitable nucleotide sequence model mtART + F + I + G4 was selected through Modeltest based on the Akaike Information Criteria (AIC) (Posada and Crandall 1998). As shown in Figure 1, the evolutionary relationship of *C*. *fasciatum* was depicted in the phylogenetic tree which was composed of two main branches. The below branch encompassed *C*. *fasciatum*, *C. umbratile,* and other species in Scyliorhinidae, indicating a closer relationship. Furthermore, the phylogenetic reconstruction supported the sister taxon of *C*. *fasciatum* and *C. umbratile* which conformed to traditional taxonomic estimates (Nakaya et al. 2013).

## Discussion and conclusion

In the present study, we sequenced and assembled the mitochondrial genome of *C. fasciatum*. We detected the genes and base compositions of the mitochondrial genome. In addition, to determine the taxonomic status of *C*. *fasciatum*, we constructed the phylogenetic tree using the maximum likelihood method on the basis of the 13 protein-coding genes of *C*. *fasciatum* and the other 15 species. The phylogenetic tree showed that the *C*. *fasciatum* had a closer relationship with species in Scyliorhinidae (Figure 1). We expect that the results of the present study will facilitate further investigations on the molecular evolution and conservation biology of *C. fasciatum.*

## Data Availability

The genome sequence data that support the findings of this study are openly available in GenBank of NCBI at https://www.ncbi.nlm.nih.gov/nuccore/MZ424309 under accession no. MZ424309.
